# Benchmarking laboratory processes to characterise low-biomass respiratory microbiota

**DOI:** 10.1038/s41598-021-96556-5

**Published:** 2021-08-25

**Authors:** Raiza Hasrat, Jolanda Kool, Wouter A. A. de Steenhuijsen Piters, Mei Ling J. N. Chu, Sjoerd Kuiling, James A. Groot, Elske M. van Logchem, Susana Fuentes, Eelco Franz, Debby Bogaert, Thijs Bosch

**Affiliations:** 1 Department of Paediatric Immunology and Infectious Diseases, Wilhelmina Children’s Hospital/University Medical Center Utrecht, 3508 AB Utrecht, The Netherlands; 2 Centre for Infectious Disease Control, National Institute for Public Health and the Environment, 3720 BA Bilthoven, The Netherlands; 3 University of Edinburgh Centre for Inflammation Research, Queen’s Medical Research Institute, University of Edinburgh, Edinburgh, EH16 4TJ UK

**Keywords:** Microbial communities, Microbiome, Next-generation sequencing, Electrophoresis

## Abstract

The low biomass of respiratory samples makes it difficult to accurately characterise the microbial community composition. PCR conditions and contaminating microbial DNA can alter the biological profile. The objective of this study was to benchmark the currently available laboratory protocols to accurately analyse the microbial community of low biomass samples. To study the effect of PCR conditions on the microbial community profile, we amplified the 16S rRNA gene of respiratory samples using various bacterial loads and different number of PCR cycles. Libraries were purified by gel electrophoresis or AMPure XP and sequenced by V2 or V3 MiSeq reagent kits by Illumina sequencing. The positive control was diluted in different solvents. PCR conditions had no significant influence on the microbial community profile of low biomass samples. Purification methods and MiSeq reagent kits provided nearly similar microbiota profiles (paired Bray–Curtis dissimilarity median: 0.03 and 0.05, respectively). While profiles of positive controls were significantly influenced by the type of dilution solvent, the theoretical profile of the Zymo mock was most accurately analysed when the Zymo mock was diluted in elution buffer (difference compared to the theoretical Zymo mock: 21.6% for elution buffer, 29.2% for Milli-Q, and 79.6% for DNA/RNA shield). Microbiota profiles of DNA blanks formed a distinct cluster compared to low biomass samples, demonstrating that low biomass samples can accurately be distinguished from DNA blanks. In summary, to accurately characterise the microbial community composition we recommend 1. amplification of the obtained microbial DNA with 30 PCR cycles, 2. purifying amplicon pools by two consecutive AMPure XP steps and 3. sequence the pooled amplicons by V3 MiSeq reagent kit. The benchmarked standardized laboratory workflow presented here ensures comparability of results within and between low biomass microbiome studies.

## Introduction

The human microbiome consists of interacting networks of microorganisms, such as bacteria, archaea and fungi. The microbial community composition varies between individuals and body sites^1–3^. To date, the gut microbiota is the most well-studied niche, and has been shown to play a vital role in human health^4–8^. However, evidence is accumulating that the microbiota in other niches such as the respiratory tract might impact human health in a similar manner^1,5,9–11^. The respiratory bacterial community is suggested to play an important role in the protection against acquisition and overgrowth of new pathogens, as well as maturation and modulation of the immune system. Additionally, there are strong indications it promotes the epithelial integrity, thereby inhibiting bacterial translocation^5,12^.

Complex microbial communities are more accurately characterised by culture-independent techniques. Especially next-generation sequencing techniques are commonly used for analysis of gut microbiota, which is a high biomass environment^1–3,5,9–14^. In contrast to the gut microbiota, the respiratory tract is less densely colonized^12,15–19^, which makes it more difficult to reliably characterise them. In particular, contaminating microbial DNA from the environment and from laboratory reagents can strongly skew bacterial profiles in low biomass materials^20–23^. Consequently, positive and negative controls are extremely important when working with low-biomass samples to correct for contamination and control for the laboratory workflow^21^. Furthermore, differences in standard operating procedures including bacterial load and the number of PCR amplification cycles have shown to affect the results significantly, making comparisons between studies more difficult^24–28^.

Therefore, a consistent workflow including suitable controls should be applied to ensure reliable microbiota analyses of low biomass materials. Here we describe the optimization of the complete laboratory process for 16S rRNA gene MiSeq library preparation protocols^2,29^. We report the effects of bacterial input, and the number of PCR cycles applied, library clean-up methods and MiSeq reagent kit chemistry on low biomass microbiota characterisation. We focus in particular on the microbial community composition of respiratory materials, which are typical low biomass samples. This study benchmarks laboratory processes to accurately characterise the microbiota of low biomass samples.

## Methods

### Study population/data collection

For the optimization experiments, we used 218 random samples collected from the nasopharynx (n = 214), oropharynx (n = 2) and saliva (n = 2) from healthy individuals (Table 1) obtained from a Dutch cross-sectional population-wide study, named Pienter-3^30^. All procedures performed were in accordance with the ethical standards of the institutional and/or national research committee. Ethical approval was granted by the national ethics committee in the Netherlands, METC Noord-Holland (METC Number: M015–022). Written informed consent was obtained from all adult participants, and parents or legal guardians of minors included in the study^30^. Following collection, saliva samples were stored in a tube containing 50% glycerol, and the upper respiratory tract samples, nasopharyngeal (NP) and oropharyngeal (OP) swabs, were stored in 1 ml of liquid Amies medium. Samples were directly frozen on dry-ice and stored at − 80 °C until further processing^30^. We used the ZymoBIOMICS microbial community standard (Zymo mock; Zymo Research, Irvine, CA, USA) and the ZymoBIOMICS microbial community DNA standard (DNA mock; Zymo Research) as positive controls.

**Table 1:** Samples and statistical method per experiment. NP = Nasopharynx, OP = Oropharynx.

Experiments	NP	OP	Saliva	Zymo mock	DNA mock	DNA blanks	NTC	Statistical method
Optimizing Zymo mock community profile				16				ANOVA-test, for global differences between groups, and Tukey's *post-hoc* test. Reference group: dilution in elution buffer. Lollipop plot
Effect of number of PCR cycles on microbial community composition		2	2					Relative abundance difference between 25, 30 and/or 35 cycles
Effect of bacterial load on microbial community composition		2	2					Relative abundance difference between 16, 125 and/or 1000 pg bacterial load
Comparing DNA concentration of NP samples with DNA blanks	214					3		Difference of DNA concentration between NP amplified by 30 PCR cycles and DNA blanks amplified by 25, 30 and 35 cycles
Concordance gel-based and AMPure XP purification	214							Bray–Curtis dissimilarity, Pearson correlation coefficient and β-coefficient
Concordance V2 and V3 MiSeq reagent kits	214							Linear model, Bray–Curtis dissimilarity, Pearson correlation coefficient and β-coefficient
Comparing low-biomass samples with DNA blanks	140					8		Unsupervised hierarchical clustering based on Bray–Curtis dissimilarity

### DNA extraction

DNA was extracted from NP swabs, OP swabs and saliva using an Agowa Mag DNA extraction kit (LGC genomics, Berlin, Germany) as previously described^29,31^, with slight modifications shown to ensure robustness for low biomass DNA extractions^29^. In each isolation run, one 200 µl aliquot of 10^3^ diluted Zymo mock was included as positive control, plus two negative controls containing lysis buffer only (referred to as DNA blanks). Samples were thawed on ice and vortexed for 10 s. Per sample, 600 µl of lysis buffer with zirconium beads (diameter 0.1 mm, Biospec Products, Bartlesville, OK, USA) and 550 µl phenol (VWR International, Amsterdam, the Netherlands) was added in a conical 1.5 ml screw-cap Eppendorf tube. Samples were mechanically disrupted twice for 2 min at 3500 oscillations/minute by bead beating (Mini-Beadbeater-24, Biospec Products) and transferred on ice for 2 min after each bead-beating step. The tubes were centrifuged for 10 min at 4500 × *g*. The clear aqueous phase was added to a 2 ml Eppendorf tube containing 1.3 ml binding buffer and 10 µl magnetic beads. After shaking for 30 min, the tubes were put in a magnetic separation rack. The supernatant was discarded, the magnetic beads were washed with wash buffer 1 and 2 and air-dried for 15 min at 55 °C. DNA was eluted in either 35 µl or 50 µl elution buffer, depending on the starting material, by shaking for 15 min at 55 °C. Supernatant was transferred to a 1.5 ml Eppendorf LoBind tube and stored at − 20 °C.

### ZymoBIOMICS microbial community standard

The Zymo mock was received from the manufacturer dissolved in DNA/RNA shield. To test the effect of dilution solvent on the generated Zymo mock profile, we prepared dilutions (10^1^–10^3^) in DNA/RNA shield, elution buffer (Qiagen, Hilden, Germany) and Milli-Q water, mimicking the DNA concentration of low biomass samples. Unless otherwise stated, we used a 10^3^ diluted Zymo mock for our analyses.

### Bacterial DNA quantification

The bacterial load was quantified by quantitative PCR (StepOnePlus Real-Time PCR System, Thermo Fisher Scientific, the Netherlands) with universal primers and probe targeting the 16S rRNA gene, containing forward primer 16S-F1 (5′-CGA AAG CGT GGG GAG CAA A-3′), reverse primer 16S-R1 (5′-GTT CGT ACT CCC CAG GCG G-3′) and probe 16S-P1 (FAM-ATT AGA TAC CCT GGT AGT CCA-ZEN) (IDT, Leuven, Belgium)^15,29^. To optimize qPCR reproducibility and to allow comparisons of DNA concentrations reliably, we developed a standard curve by using a synthesized fragment of the 16S rRNA gene (gBlocks Gene Fragment, IDT, 5′-CGG TGC GAA AGC GTG GGG AGC AAA CAG GAT TAG ATA CCC TGG TAG TCC ACG CCG TAA ACG ATG TCT ACT AGC TGT TCG TGG TCT TGT ACT GTG AGT AGC GCA GCT AAC GCA CTA AGT AGA CCG CCT GGG GAG TAC GAA CGC AAG-3′).

### MiSeq library preparation and sequencing

The V4 region of the 16S rRNA gene was amplified by PCR using the 515F (5′-GTG CCA GCM GCC GCG GTA A-3′) and 806R (5′-GGA CTA CHV GGG TWT CTA AT-3′) primers including the Illumina adapters and sample specific barcodes^2,32,33^. Each 25 µl PCR reaction consisted of 0.5 µl Phusion Hot Start II High-Fidelity DNA Polymerase, 5 µl 5 × Phusion HF Buffer (Thermo Fisher Scientific), 7 µl HPLC grade water (Instruchemie, Delfzijl, the Netherlands), 2.5 µl of 2 mM dNTP mix (Roche, Mannheim, Germany), 5 µl of 5 µM barcoded primer 515F, 5 µl of 5 µM barcoded primer 806R and 5 µl template DNA. PCR reactions were executed using the following successive steps; 98 °C for 30 s; 30 cycles at 98 °C for 10 s, 55 °C for 30 s and 72 °C for 30 s and a final hold of 5 min at 72 °C. Samples with a 16S rRNA gene DNA concentration of < 20 pg/µl (< 100 pg input DNA) were used undiluted, samples with a higher concentration were diluted in HPLC grade water, accordingly. To study the effect of PCR conditions on the microbiota profile, 16, 125 and 1000 pg of bacterial load from two OP and two saliva samples were amplified using 30 cycles. The input DNA of 125 pg was additionally, separately, amplified by 25 and 35 PCR cycles, respectively. DNA blanks, no template controls (NTC), Zymo mocks and DNA mocks were included in each PCR plate and sequenced alongside the samples. The fragment size of the amplicon was assessed using agarose gel electrophoresis and quantified by Quant-iT PicoGreen dsDNA Assay Kit (Thermo Fisher Scientific). Barcoded amplicons were pooled in equimolar ratios. To study the optimal purification method, we purified the pool with two different cleaning methods; 1. agarose gel purification, extracting the DNA using GeneJET Gel Extraction and DNA Cleanup Micro Kit (Thermo Fisher Scientific), and subsequent purification by 0.9 × AMPure XP magnetic beads (Beckman Coulter, the Netherlands), or 2. by two consecutive purifications using 0.9 × AMPure XP. The library was prepared as recommended by Illumina and sequenced using the MiSeq reagent kit V2 or V3 (paired end, 500 bp) on an Illumina MiSeq instrument (Illumina Inc., San Diego, CA, US).

### Data analysis

All sample libraries were simultaneously processed using an in-house bioinformatics pipeline^1,3,11,34,35^. First, we performed adaptive window trimming with a quality threshold of Q30, retaining those reads with a minimum length of 150 nucleotides (Sickle, version 1.33)^36^. Sequencing errors were reduced by an error correction algorithm (BayesHammer, SPAdes genome assembler toolkit, version 3.12.0). Paired-end sequenced reads were assembled into contigs using PANDAseq (version 2.10) and demultiplexed using QIIME (version 1.9.1)^38,39^. Singleton sequences and chimeras were removed (UCHIME; implemented in the VSEARCH toolkit v2.0.3). VSEARCH abundance-based greedy clustering was performed to pick OTUs (operational taxonomic unit) with a 97% identity threshold^40^. OTUs were taxonomically annotated by the Naïve Bayesian RDP classifier using the SILVA 119 release reference database^41,42^. OTUs were assigned a rank number based on their abundance across the total dataset.

Analyses were performed in R version 4.0.2 within R studio version 1.4.623. OTU read counts were normalised using total sum scale resulting in relative abundances of OTUs. Microbiota profiles were visualized using stacked bar charts/boxplots. Lollipop plots were used to visualize the differences in relative abundance of each OTU between sequenced diluted Zymo mocks and the theoretical Zymo mock. To assess overall differences in microbial community composition, including low and high abundant OTUs, between (pairs of) samples we used Bray–Curtis dissimilarity matrix, where zero indicates an identical composition between pairs. Non-metric multidimensional scaling (NMDS) based on the Bray–Curtis dissimilarity matrix was used to visualize differences in microbial profiles between low-biomass samples and DNA blanks^1^. We investigated the minimal DNA concentration for reliant microbiome analyses by comparing the microbial profiles of DNA blanks and low-density samples using an unsupervised hierarchical clustering approach based on the Bray–Curtis dissimilarity matrix, which was illustrated in a dendrogram. Silhouette and Calinski-Harabasz indices were used to determine the optimal number of clusters^1^. To assess the impact of MiSeq reagent kits/purification methods, we determined the Pearson correlation of log_10_ + 1-transformed relative abundances of OTUs with > 0.1% abundance in at least 20 samples. To test for significant differences in Zymo mock composition with different dilution solvents we used an ANOVA-test with Tukey's post hoc test to determine statistical significance between specific groups. Linear models were used to calculate the statistical significance between the number of reads per sample sequenced by V2 and V3 reagent kits. A p-value < 0.05 was considered significant.

## Results

### DNA extraction

#### Zymo mock dilution optimization

**Figure 1:**
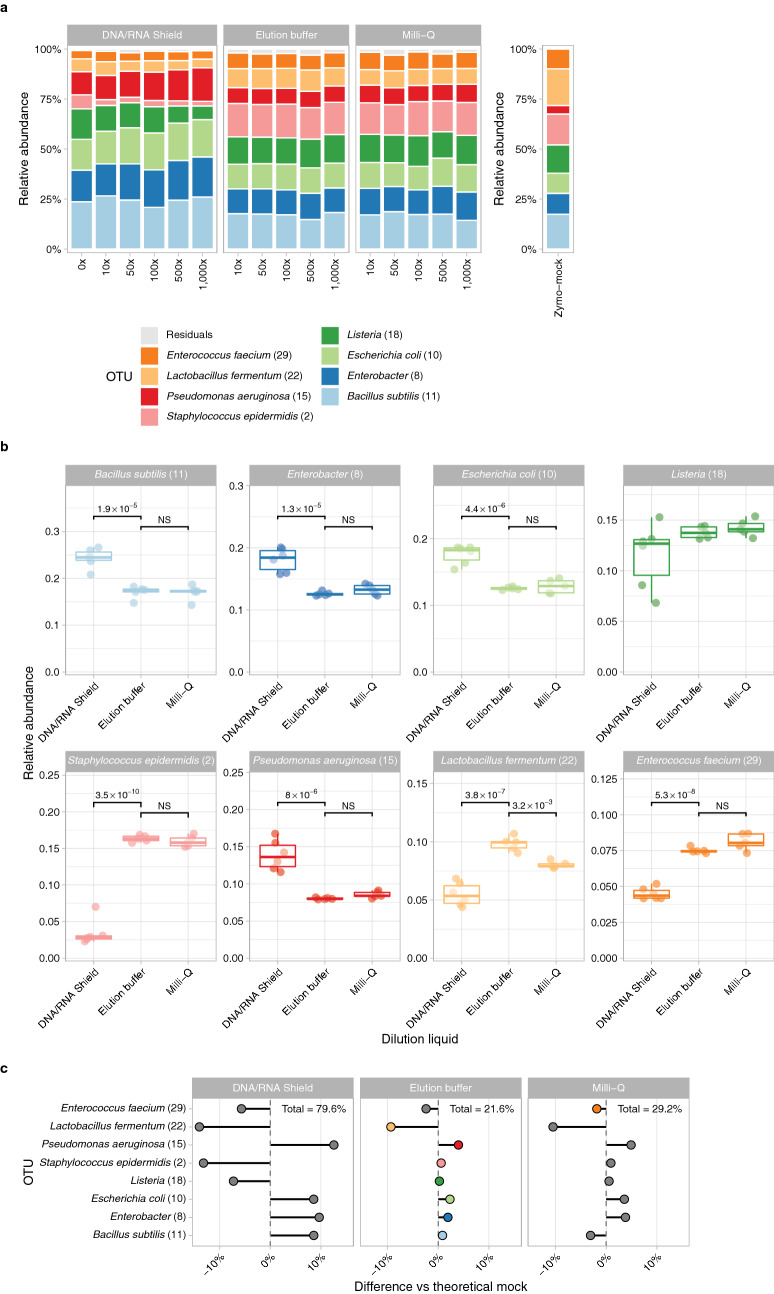
Bacterial composition of Zymo mock diluted in DNA/RNA shield (n = 6; undiluted and 10^1^–10^3^ × diluted), elution buffer (n = 5; 10^1^–10^3^ × diluted) and Milli-Q (n = 5; 10^1^–10^3^ × diluted). (**a**) Stacked bar charts show the relative abundance of the top 8 operational taxonomic units (OTUs) in (un)diluted Zymo mock stratified by dilution solvent and the theoretical undiluted Zymo mock composition. The diluted Zymo mocks are annotated in the bioinformatic pipeline and have different annotations than the OTUs of the theoretical Zymo mock. Based on inspection of community profiles we found that the OTU annotated as *Salmonella enterica* refers to *Enterobacter*, *Listeria monocytogenes* to *Listeria*, *Staphylococcus aureus* to *Staphylococcus epidermidis* and *Enterococcus faecalis* to *Enterococcus faecium.* (**b**) Boxplots show the relative abundance of each OTU in the dilution solvents. Boxplot depicts the 25th and 75th percentiles by lower and upper hinges, respectively, the median is depicted by a horizontal line in the box. The measurements that fall within 1.5 times the interquartile range are shown by whiskers. Statistical significance in relative abundance between dilution solvents were assessed by ANOVA test. A Tukey's post hoc test was used to determine statistical significance between elution buffer and DNA/RNA shield or Milli-Q. (**c**) Lollipop plot shows the differences in relative abundance of each OTU between the 10^3^ × diluted Zymo mocks and the theoretical Zymo mock. A strong positive value indicates a higher relative abundance of this OTU found than expected based on the theoretical mock and a strong negative value means that there is less of this OTU observed in the diluted Zymo mock. Coloured points indicate the lowest difference compared to the theoretical Zymo mock for that specific OTU across the dilution solvents. The percentage demonstrates the total cumulative absolute difference in relative abundance across the 8 OTUs compared to the theoretical mock.

To mimic the concentration of low-biomass samples, a Zymo mock dilution series (10^1^–10^3^ ×) was prepared. Zymo mocks were diluted in DNA/RNA shield (n = 6), elution buffer (n = 5) and Milli-Q (n = 5). Dilution in DNA/RNA shield resulted in a significantly different microbiota profile in comparison to elution buffer and Milli-Q across dilutions (Fig. 1a and b), which also deviated most from the theoretical Zymo mock profile. We observed an overrepresentation of *Bacillus subtilis (11), Enterobacter (8), Escherichia coli (10)* and *Pseudomonas aeruginosa (15)* and an underrepresentation of *Staphylococcus epidermidis (2), Lactobacillus fermentum (22)* and *Enterococcus faecium (29)* in Zymo mocks diluted in DNA/RNA shield compared to elution buffer (Fig. 1b). In contrast, when comparing dilution in Milli-Q versus elution buffer, we observed a significant difference in *Lactobacillus fermentum (22)* abundance (median 7.9% vs 10.0%, respectively, p-value < 0.001). The Zymo mock diluted in elution buffer most closely resembled the theoretical Zymo mock composition (Fig. 1c). Therefore, for further experiments, we continued with elution buffer as dilution solvent.

### Library preparation

#### Influence of PCR amplification cycles and bacterial density on the microbiota profile

**Figure 2:**
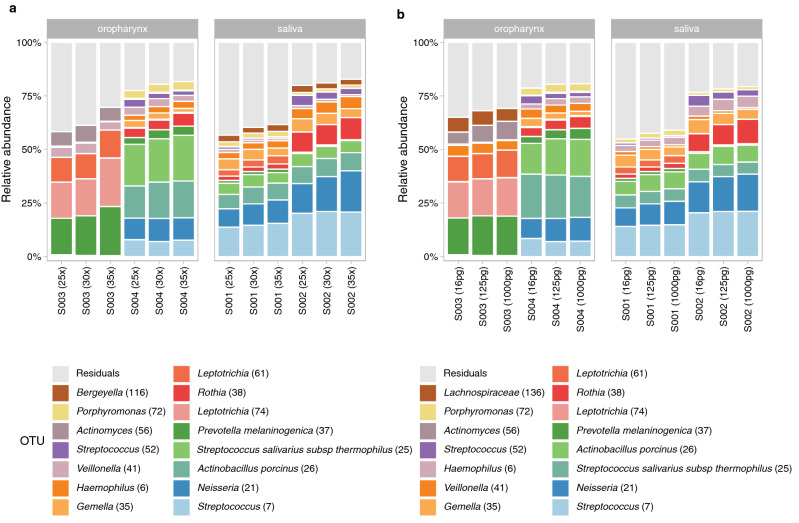
Microbiota composition profiles of 2 oropharynx (S003 and S004) and 2 saliva samples (S001 and S002). Stacked bar charts of the relative abundance of the top 15 OTUs and the residuals are shown. (**a**) Microbiota profile of OP and saliva samples amplified by 25, 30 or 35 PCR cycles. (**b**) Microbiota composition of OP and saliva samples with a bacterial load of 16, 125 or 1000 pg.

**Figure 3:**
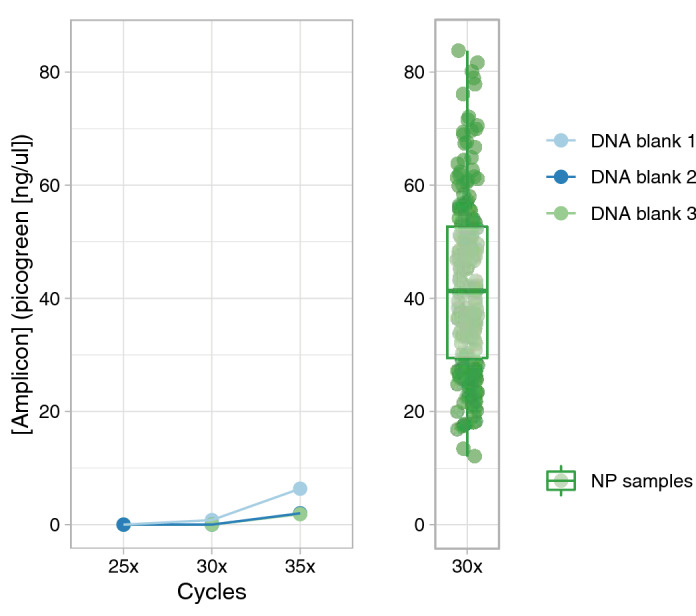
DNA concentration of 3 DNA blanks and 214 NP samples after PCR amplification. DNA concentration was estimated by picogreen quantification. The concentration of DNA blanks is visualized in the left graph shown for 25, 30 and 35 amplification cycles. The DNA concentration of the NP samples amplified by 30 PCR cycles are shown in the boxplot. NP samples had a median concentration of 41.3 ng/µl (range: 12.1–83.7). Boxplot depicts the 25th and 75th percentiles by lower and upper hinges, respectively, the median is depicted by a horizontal line in the box. The measurements that fall within 1.5 times the interquartile range are shown by whiskers. Each green dot is an individual NP sample.

Next, we tested the effect of the number of PCR amplification cycles on the microbial community profile. To this end, 125 pg of microbial DNA of 2 OP and 2 saliva samples, were amplified using 25, 30 and 35 PCR cycles. We observed that a higher number of PCR cycles resulted in minor increases in relative abundance of especially high abundant OTUs. The abundance of *Neisseria (21)* (8.6/13.9%, 10.0/16.3% and 10.9/19.2% for 25, 30 and 35 cycles, respectively) increased in both saliva samples with increasing PCR cycles (Fig. 2a). One OP sample showed a higher relative abundance of *Prevotella melaninogenica (37)* (17.0%, 18.4% and 22.9% for 25, 30 and 35 cycles, respectively) and *Leptotrichia (74)* (16.8%, 17.3% and 22.6% for 25, 30 and 35 cycles, respectively) with increasing PCR cycles. However, a higher number of PCR cycles also resulted in an increased amplification of DNA in blanks (Fig. 3). Given the increased risk of contamination bias when using 35 PCR cycles on the one hand, and higher rate of amplification failures when using 25 PCR cycles on the other hand (data not shown), we therefore recommend an optimal number of PCR amplification cycles of 30.

To assess the effects of bacterial load on microbial community profiles, we tested three different quantities of bacterial input DNA (16, 125 and 1000 pg) of 2 saliva and 2 OP samples. We noticed that increasing DNA concentrations modestly affect the relative abundance of high abundant OTUs (Fig. 2b). In 3 of the 4 samples, we observed a modest increase in the relative abundance of *Neisseria (21)* (9.4/8.7/14.5%, 10.8/10.0/16.3% and 11.1/11.0/17.4% for 16, 125 and 1000 pg, respectively). Another OP sample showed modest increased relative abundance of *Leptotrichia (74)* with increasing template input (16.8%, 17.3% and 17.9% for 16, 125 and 1000 pg, respectively). Despite minor differences, we propose to standardise to a bacterial load of 125 pg as input DNA for MiSeq PCR in case of low-biomass samples, given that many low biomass samples do not meet a 1000 pg yield threshold.

#### Concordance between library clean-up methods

**Figure 4:**
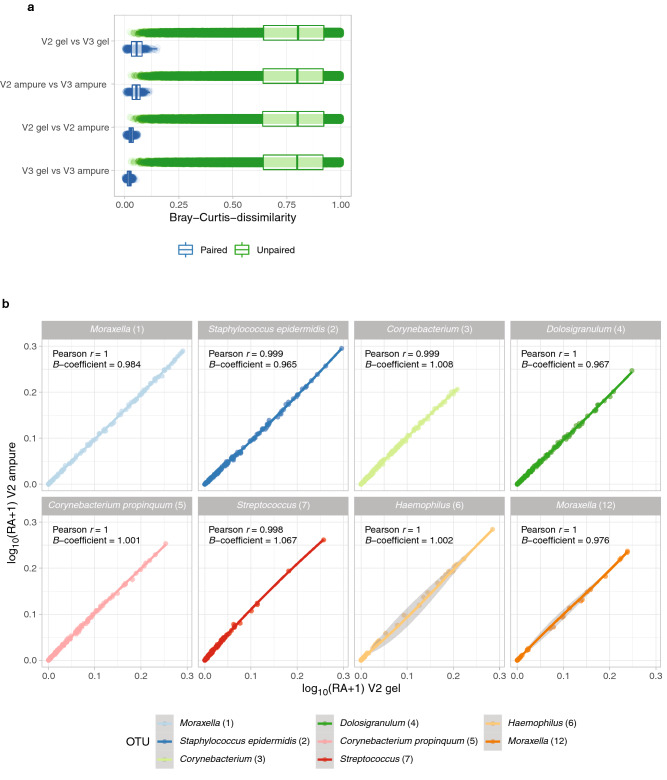
Similarity of a library (n = 214) purified by gel or AMPure XP. (**a**) To quantify differences in the overall microbial community composition between (pairs of) samples, Bray–Curtis dissimilarity was used, where zero indicates an identical composition between pairs. Unpaired dissimilarity was determined by calculating the dissimilarity of a given sample to all other (unpaired) samples in the other group, whereas paired dissimilarity refers to the dissimilarity between pairs of samples in both groups. Boxplot depicts the 25th and 75th percentiles by lower and upper hinges, respectively, the median is depicted by a horizontal line in the box. The measurements that fall within 1.5 times the interquartile range are shown by whiskers. (**b**) Correlation plots visualizes log_10_ + 1-transformed relative abundances of the top 8 OTUs of a pool sequenced using the V2 reagent kit, comparing gel-based and AMPure XP purification methods. For each OTU, the Pearson correlation coefficient and regression coefficient (slope) was calculated. Both the correlation coefficient and the slope show a value close to 1.0, indicating a perfect correlation between purification methods for these OTUs.

To further optimize our workflow, we studied the influence of the gel-based purification and the AMPure XP clean-up on the eventual microbiota profile, by purifying an amplicon pool containing 214 samples using both procedures (Table 1). The obtained microbiota profiles per sample were highly similar between methods (paired Bray–Curtis dissimilarity median: 0.03; range: 0.0–0.06), indicating a high concordance between both clean-up methods (Fig. 4a). Furthermore, we compared the relative abundances of the top 8 OTUs per sample and observed a correlation and regression coefficient of ~ 1.0 for all OTU abundances observed by both methods (Fig. 4b), indicating a near perfect concordance, and thus negligible differences between the tested library clean-up methods. Following, we chose to continue with the AMPure XP purification method as it is faster compared to gel-based purification.

### MiSeq sequencing

#### Concordance between the V2 and V3 MiSeq reagent kits

**Figure 5:**
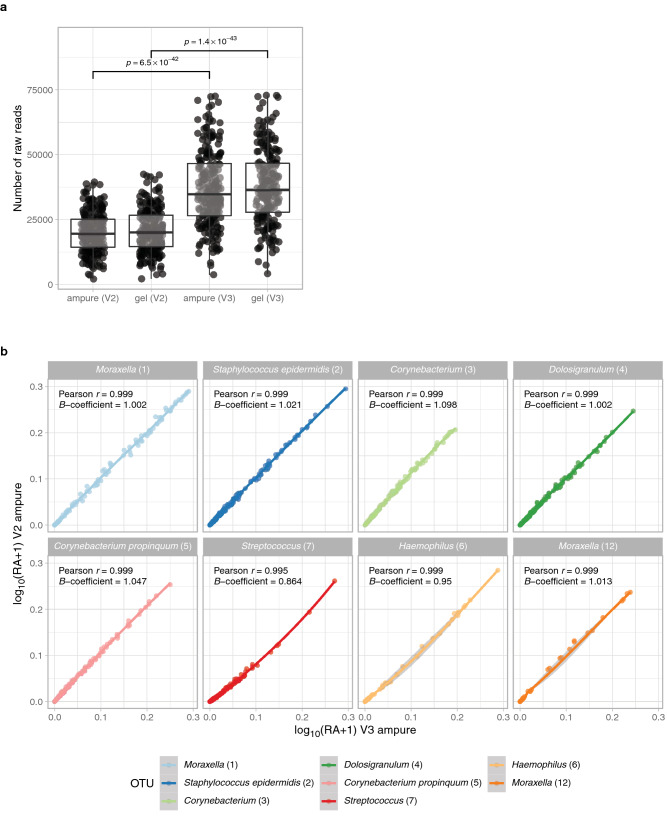
Similarity of a library (n = 214) sequenced by MiSeq V2 and V3 kits. (**a**) Number of reads of samples sequenced by V2 and V3 kit stratified by purification method. Statistical significance in number of reads between V2 and V3 kit was calculated by a linear model. (**b**) Correlation plots visualize log_10_ + 1-transformed relative abundances of the top 8 OTUs of a pool purified by AMPure XP, comparing the V2 or V3 reagent kit. For each OTU, the Pearson correlation coefficient and regression coefficient was calculated. Both the correlation coefficient and almost all slopes show a value close to 1.0, indicating a near perfect correlation between purification methods for these OTUs.

To study the concordance between the V2 and V3 MiSeq reagent kits, we used the same set of samples as described when validating the library clean-up methods (Table 1). The mean number of reads per sample purified by AMPure XP was significantly different between the V2 and V3 kit (p-value < 0.001), with 20,060 (range: 2123–39486) versus 36,981 reads per sample (range: 3781–72469 reads), respectively (Fig. 5a). The overall microbial community profile only marginally differed between both sequencing methods, as indicated by the very high similarity observed between paired samples (Bray–Curtis dissimilarity median: 0.05; range: 0.0–0.1) when compared to unpaired samples (Bray–Curtis dissimilarity median: 0.8; range: 0.03–1.0) (Fig. 4a). Additionally, we compared the relative abundances of the top 8 OTUs and observed a correlation coefficient of ~ 1.0 for all those OTUs and a regression coefficient of ~ 1.0 for 7 of those OTUs (Fig. 5b), with *Streptococcus (7)* slightly underrepresented in the V2 kit (regression coefficient: 0.9). For lower prevalent OTUs the variance in data was too large, to reliably conclude on similarity of data. We conclude that given the high concordance between MiSeq reagent kits, we prefer to use the more recent V3 MiSeq kit, as it yields a higher number of reads per sample.

#### Microbiota profiles of low biomass samples compared to DNA isolation blanks

**Figure 6:**
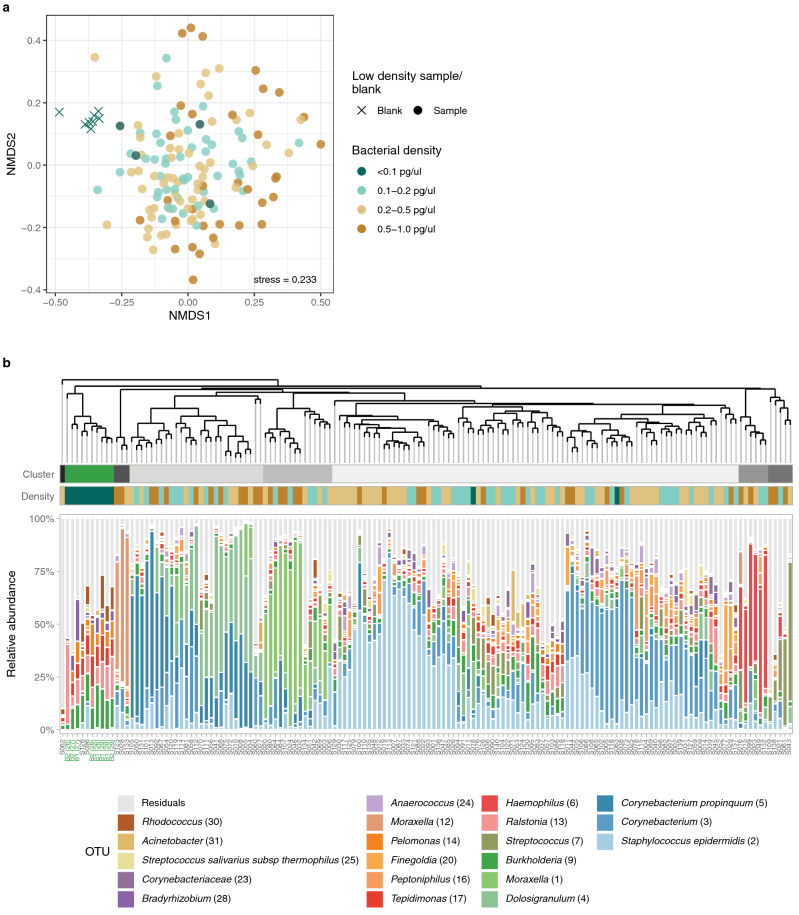
Microbiota profiles of nasopharyngeal (NP) samples (n = 140) and DNA blanks (n = 8). (**a**) Two-dimensional nonmetric multidimensional scaling (NMDS) plot, based on the Bray–Curtis dissimilarity matrix, visualizes the microbial community composition (each point) of the NP samples and DNA blanks (cross). Samples are stratified by DNA concentration based on 16S rRNA gene qPCR quantification (concentration pg/µl: < 0.1; 0.1–0.2; 0.2–0.5; 0.5–1.0). The stress value illustrates how well the high-dimensional data are captured in the two-dimensional space; a value of around 0.2 indicates an acceptable representation. (**b**) Dendrogram visualizes an average linkage hierarchical clustering of NP samples and DNA blanks based on the Bray–Curtis dissimilarity index. The length of the branches represents the similarities between samples and DNA blanks. Stacked bar charts show the relative abundance of the top 20 OTUs and the residuals. Based on the clustering indices, 8 clusters were identified, 7 clusters solely consisted of NP samples (grey) and one separate cluster contained all DNA blanks and 2 NP samples (in green). Bacterial density colours correspond with colours used in the NMDS plot.

We finally tested whether the microbial community profiles of very low biomass samples could be distinguished from procedural blanks, using a range of low biomass samples. When comparing the microbiota profiles of 140 NP samples (range: 0.06–1.00 pg/µl) and 8 DNA blanks (0.02–0.07 pg/µl) (Table 1), we found that the blanks still clustered away from the NP samples (Fig. 6a). Using an unsupervised hierarchical clustering of both samples and blanks, we identified 8 different clusters, 7 clusters containing exclusively NP samples and one cluster containing DNA blanks and 2 NP samples (Fig. 6b). These 2 NP samples had a concentration lower than 0.10 pg/µl, while the other 2 NP samples with < 0.10 pg/µl clustered with all other NP samples containing > 0.10 pg/µl. Therefore, we advise to only use samples for DNA amplification and sequencing with a minimum concentration of 0.10 pg/µl, or a threshold slightly above the blanks in case local signals observed in DNA blanks are higher. Although, low biomass samples may still contain contaminating DNA, these samples can be clearly distinguishable from DNA blanks and are more likely to still elicit sufficient reads after consecutive bioinformatic clean-up.

## Discussion

To study high biomass fecal microbiota, Costea et al. recommended the use of a standardized protocol to ensure reproducibility and comparibility among studies^43^. Here, we show the importance of a standardized DNA extraction and sequencing protocol for low biomass samples like respiratory materials as well. The samples used for this project consist of a large number of NP samples (n = 214) with a range of (low biomass) bacterial loads. Positive and negative controls were included during DNA extraction, MiSeq PCR, sequencing and in the bioinformatic pipeline. Hereby, we could study the accurate processing of DNA for 16S rRNA gene sequencing and the limitations of working with low biomass samples. Noteworthy, the library clean-up methods (gel-based purification or AMPure XP), and the MiSeq reagent kits (V2 or V3 chemistry), resulted in modest to no effects on overall microbial community profiles.

We compared the labour-intensive gel-based size selection and a column-based clean-up method (AMPure XP), which can select for DNA size in a fast and effective manner^44–47^. A specific ratio of 0.9 × AMPure XP leads to minimal loss of library DNA concentration and complete removal of primer dimers^48^. Microbial community profiles of samples processed using each of these two methods in parallel showed high similarity. Furthermore, we observed a near perfect concordance between relative abundances of the 8 most abundant OTUs in paired analyses. Since the different cleaning procedures elicited highly similar microbial profiles, we propose to use AMPure XP for fast library clean-up.

In a whole genome sequencing study, the microbiota data obtained by whole genome shotgun sequencing using the V2 (2 × 150 bp) and V3 (2 × 300 bp) MiSeq reagent kits showed already to be highly similar^49^. We are the first to compare the microbiota data of a 16S rRNA gene pool sequenced with the same settings using the V2 and V3 reagent kits (2 × 250 bp). We observed a very high concordance between the V2 and V3 kit; the modest underrepresentation of *Streptococcus* in the V2 kit is likely a result of differences in number of freeze–thaw cycles (one cycle difference) of the library in our study, rather than differences in kits used^50^. To understand the ecology of the respiratory microbiome, it is critical to study the whole microbiome including the low abundant bacteria^49^, which underlines the importance of sufficient sequencing depth. Here, we noticed that the sequencing depth per sample almost doubled when we sequenced using the V3 kit. Furthermore, the V3 MiSeq reagent kit offers an increased cluster density, higher read length and improved quality scores, thus being preferable above the V2 kit.

The inclusion of negative controls is vital to accurately study the microbiota^20–23^. Contaminants can have a significant impact on the microbial data of low biomass samples^21^. Though not a primary research question in our study, we confirmed that samples with a concentration as low as 0.1 pg/µl can be consistenly amplified and show a microbiota composition that is distinguishable from the DNA extraction blanks, even without removing the contaminanting OTUs in the bioinformatic pipeline. Discrimination between samples and blanks should further improve when using dedicated bioinformatic tools^51,52^ such as the *decontam* R-package, which allows for the identification and removal of contaminating OTUs, ideally based on a large number of negative controls^51^. DNA extraction blanks and ‘no template’ controls will therefore not only help to identify limits within laboratory protocols, but also help to control for contaminating DNA in downstream analyses.

We demonstrated that the bacterial profile of the Zymo mock, when diluted, can be influenced by the solvent used (DNA/RNA shield, MilliQ and elution buffer). Sample storage should therefore also be optimised for the positive controls. Dilution of Zymo mock in elution buffer most closely resembled the bacterial profile of the theoretical mock, and therefore seems preferable.

Several studies have described the effect of PCR conditions on the microbial community profile. A higher number of PCR cycles has shown to lead to an increased concentration of contaminating DNA, point mutation artifacts and chimera formation^21,24,25,27,28^. An increased number of PCR cycles will also lead to a higher concentration of contaminating DNA in blanks and low biomass samples. Given our focus on low biomass samples, we find 30 PCR cycles to be optimal, allowing for sufficient amplicon yield, yet still limiting the impact of contaminating DNA. An initial bacterial input of 16 pg is feasible for most of the NP samples used in this study, though more samples would have to be diluted, resulting in a higher amplification of contaminating DNA and biased microbial profiles^21^. We here demonstrated that varying template DNA concentration and PCR cycles resulted in minor differences in the microbiota profile. Eventually, 30 amplification cycles with a bacterial DNA input of 125 pg resulted in sufficient amplicon concentrations for MiSeq sequencing and low background contamination.

This study has several strengths. We improved the laboratory processes by optimizing several components of our workflow, e.g. clean-up methods and PCR conditions. This resulted in an optimized MiSeq protocol for analysis of low-biomass samples. We used diluted positive controls to mimic the concentration of low biomass samples and studied the influence of dilution solvents on the bacterial profiles of these positive controls. To characterise the influence of potential reagent and environmental contamination, we included appropriate negative controls, which are extremely important when studying low biomass samples. We compared the libraries sequenced by different MiSeq reagent kits (V2 and V3) with the same MiSeq settings (2 × 250 bp). Our study also has some limitations. Despite the advantages of the Zymo mock as a positive control, it only contains few respiratory bacteria and represents low microbial diversity. Preferably, we would like to use a mock which mimics the microbiota composition of NP samples, has a more diverse profile and consists of different ratios of bacteria. This is something to consider for individual laboratories to introduce when they decide to focus on low biomass samples. Furthermore, we did not include a sufficient number of Zymo mocks to test whether different PCR conditions and different MiSeq reagent kits have an influence on the Zymo mock profile. In addition, there are other laboratory factors that may also impact microbiota results, including different types of polymerases^53,54^, which were outside the scope of the current study.

## Conclusion

In this study, we demonstrated the reliability of our DNA extraction and 16S rRNA gene MiSeq library preparation protocol for low biomass samples. Template concentration and number of PCR cycles had a modest influence on the microbiota profiles, while the PCR purification method and MiSeq sequencing kit had no significant effects on the microbial profiles. Therefore, we propose to use samples with a DNA concentration of 0.1–20 pg/µl which can be amplified with 30 PCR cycles. After pooling, the library can be purified by two consecutive 0.9 × AMPure XP purification steps and sequenced with the V3 MiSeq reagent kit. We confirmed that even extremely low biomass samples can be distinguished from DNA blanks. We here present a benchmarked standardized laboratory workflow that, when consistently and more widely used, ensures comparability of results within and between studies. In addition, the workflow could be useful to study the microbiota of other low biomass samples, e.g. lung, skin, blood, but also environmental samples in a standardized way.

## Data Availability

Sequence data that support the findings of this study have been deposited in the National Center for Biotechnology Information Sequence Read Archive database with BioProject ID PRJNA718293.
